# Blockade of autophagy reduces pancreatic cancer stem cell activity and potentiates the tumoricidal effect of gemcitabine

**DOI:** 10.1186/s12943-015-0449-3

**Published:** 2015-10-12

**Authors:** Ming-Chen Yang, Hao-Chen Wang, Ya-Chin Hou, Hui-Ling Tung, Tai-Jan Chiu, Yan-Shen Shan

**Affiliations:** Department of Microbiology and Immunology, College of Medicine, National Cheng Kung University, Tainan, Taiwan; Institute of Clinical Medicine, College of Medicine, National Cheng Kung University, Tainan, Taiwan; Department of Surgery, National Cheng Kung University Hospital, Tainan, Taiwan; Department of Medical Oncology, Kaohsiung Chang Gung Memorial Hospital, Institute of Clinical Medical Sciences, Chang Gung University College of Medicine, Kaohsiung, Taiwan

**Keywords:** ALDH1, Autophagy, Cancer stem cells, CD133, CD44, Gemcitabine, Osteopontin, Pancreatic cancer

## Abstract

**Background:**

Cancer stem cells (CSCs) are considered responsible for the recurrence and chemoresistance of cancer. Dysregulated autophagy is highly prevalent in many types of cancer including pancreatic cancer and has been implicated in cytoprotection and tumor promotion. This study aimed to investigate the role of autophagy in regulating cancer stemness and chemoresistance of pancreatic cancer.

**Methods:**

The correlation between autophagy and CSCs and its clinical significance were analyzed using pancreatic cancer tissue microarrays. Genetic and pharmacological approaches were applied to explore the function of autophagy on CSC activity and gemcitabine resistance of pancreatic cancer cells *in vitro* and *in vivo*.

**Results:**

LC3 expression positively correlated with the expression of CSC markers aldehyde dehydrogenase 1 (ALDH1), CD44, and CD133 in pancreatic cancer tissues. High coexpression of LC3/ALDH1 was associated with both poor overall survival and progression-free survival. In pancreatic cancer cell lines, higher LC3-II expression was observed in the sphere-forming cells than in the bulk cells. Blockade of autophagy by silencing *ATG5*, *ATG7*, and *BECN1* or the administration of autophagy inhibitor chloroquine markedly reduced the CSC populations, ALDH1 activity, sphere formation, and resistance to gemcitabine *in vitro* and *in vivo*. Furthermore, osteopontin (OPN) was found to stimulate LC3-II, ALDH1, CD44, and CD133 expression in PANC-1 cells, whereas this effect could be prevented by OPN knockdown and autophagy blockade. After treatment with various inhibitors against the major signaling pathways downstream of OPN, only the inhibitor of NF-κB activation, BAY 1170–82, could effectively counteract OPN-induced autophagy and CSC activity. According to the histochemical results, pancreatic cancer patients manifesting high levels of OPN/LC3/ALDH1 and OPN/CD44/CD133 had poor survival.

**Conclusions:**

Induction of autophagy mediated by OPN/NF-κB signaling is required for maintenance of pancreatic CSC activity. Combination of gemcitabine with pharmacological autophagy inhibitors is a promising therapeutic strategy for pancreatic cancer.

**Electronic supplementary material:**

The online version of this article (doi:10.1186/s12943-015-0449-3) contains supplementary material, which is available to authorized users.

## Background

The cancer stem cell (CSC) theory postulates that tumors may arise from a small fraction of cancer cells that are response for tumor initiation and maintenance [1]. The high metastatic potential and intense resistance to chemotherapy and radiation therapy in several cancers have been linked to CSCs, revealing new opportunities for cancer treatment [2–4]. Pancreatic CSCs have been identified by several putative markers, such as CD44, CD24, EpCAM, CD133, and aldehyde dehydrogenase 1 (ALDH1) [5–7]. The presence of CSCs in pancreatic cancer was associated with poor patient outcomes [8]. Pancreatic tumors containing higher percentage of CSCs displayed enhanced chemoresistance and metastatic activity [6, 9, 10]. Accordingly, therapies that selectively target CSCs may offer a greater promise for pancreatic cancer treatment.

Autophagy is a cellular self-degradation process that is required to maintain cell homeostasis. Alterations in autophagy-related signaling pathways frequently occur in many human diseases, including cancer [11]. Considerable evidence has supported that autophagy maintains the survival of cancer cells through conferring stress tolerance and limiting damages [12]. In pancreatic cancer, sustained activation of autophagy is associated with poor survival [13]. Genetic or pharmacologic abrogation of autophagy suppressed the growth of pancreatic cancer cells *in vitro* and led to tumor regression *in vivo* due to autophagy inhibition-mediated reactive oxygen species production, DNA damages and altered cell metabolism [14]. Therefore, autophagy is required for pancreatic cancer progression. Because autophagy acts as a survival pathway in cells under stress, much attention has been paid to the role of autophagy in CSC biology. Genetic inhibition of autophagy reduced the proportion of breast cancer cells bearing a CD44^+^/CD24^-/low^ CSC-like phenotype, suggesting the role of autophagy in maintaining the typical breast CSCs [15]. Blockade of both autophagy flux and lysosomal proteolyic activity by K^+^/H^+^ ionophore Salinomycin effectively reduced the population of ALDH^+^ breast CSCs [16]. Treatment with the autophagy inhibitor chloroquine (CQ) strongly promoted γIR-induced cell death in highly radioresistant patient-derived stem-like glioma cells [17]. In pancreatic cancer cells, high levels of autophagy have been observed under basal conditions [14, 18]; however, the relation between autophagy and pancreatic CSCs remains to be explored.

Osteopontin (OPN), a secreted glycoprotein, has been implicated in a variety of physiological and pathophysiological processes, such as bone remodeling, angiogenesis, immunity, atherosclerosis, and cancer progression [19, 20]. By interacting with CD44 family of receptors or integrin αvβ3, OPN can activate several downstream signaling pathways, such as PI3K/AKT, NF-κB, and MEK/ERK [21]. OPN overexpression in many types of cancer has been considered a poor prognostic marker [22]. Recently, increased OPN expression has been observed in sphere-growing stem-like cells of pancreatic cancer compared with their adherent counterpart [23]. OPN overexpression significantly increased the formation of spheres derived from the brain tumor cells of p53/PTC double heterozygous mice [24], suggesting a role of OPN in regulating CSC activity. Given that OPN can induce autophagy directly through integrin/CD44 and p38 MAPK-mediated pathways in vascular smooth muscle cells [25], we sought to investigate whether OPN can increase pancreatic CSC activity through stimulation of autophagy.

## Results

### CSC markers colocalize with the autophagy protein LC3 in pancreatic cancer cells

**Fig. 1:**
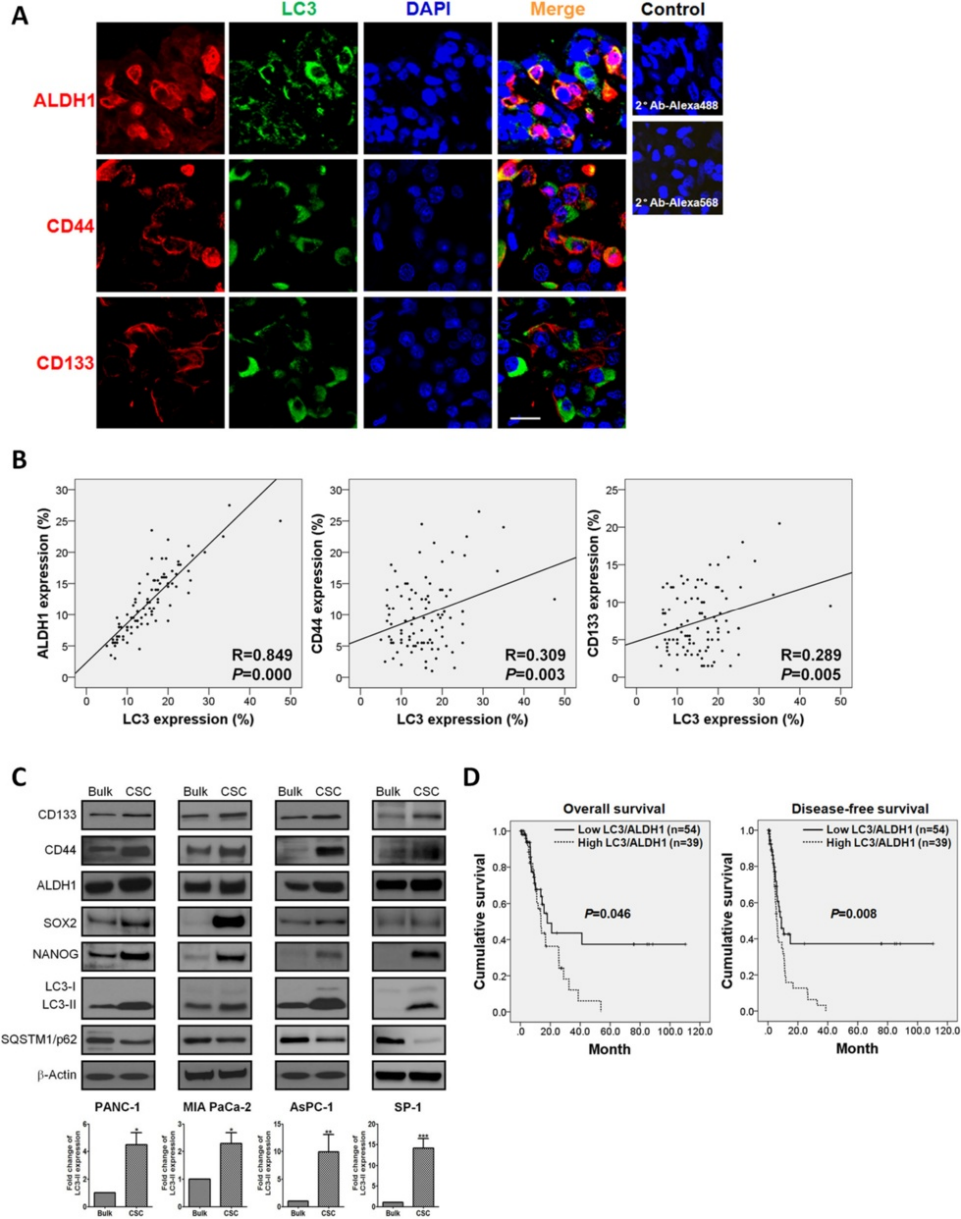
Autophagy marker LC3 expression correlates with CSC marker expression in pancreatic cancer cells. **a** Pancreatic tumor tissues were immunofluorescently stained for autophagy marker LC3 (green) and CSC markers ALDH1, CD44, and CD133 (red). Samples stained with secondary antibodies alone served as a negative control. Magnification: 800×, scale bar: 20 μm. **b** Immunofluorescence analysis of LC3, ALDH1, CD44, and CD133 was performed in TMAs. LC3 expression showed a significant positive correlation with ALDH1 (*R* = 0.849, *P* < 0.001), CD44 (*R* = 0.309, *P* = 0.003), and CD133 expression (*R* = 0.289, *P* = 0.005). **c** PANC-1, MIA PaCa-2, AsPC-1, and SP-1 cells were cultured in ultra-low attachment plates for 14 days to form spheres. The lysates of sphere-forming cells were collected for Western blotting with the indicated antibodies. β-Actin was used as a loading control. The levels of LC3-II were normalized against β-Actin. Values represent the means ± SE of three independent experiments. *, *P* < 0.05; **, *P* < 0.01; ***, *P* < 0.001, *vs.* bulk cells. **d** Kaplan–Meier analysis showed that high levels of LC3/ALDH1 were significantly associated with poor OS (*P* = 0.046) and DFS (*P* = 0.008)

To determine the relationship between autophagy and CSCs, we performed an immunofluorescence study in tissue microarrays (TMAs) of 93 pancreatic tumors and calculated the correlation coefficients between LC3 and CSC marker expression. Autophagy was demonstrated by LC3 puncta in cells expressing ALDH1, CD44, and CD133 (Fig. 1a). LC3 colocalized with LAMP1, a lysosomal marker used for detection of LC3^*+*^*/*LAMP1^*+*^ autolysosome formation [26], in pancreatic tumor tissues, and SQSTM1/p62, an autophagy marker that is degraded during autophagy [26], was weakly stained in cells expressing LC3, revealing the activation of autophagy in pancreatic cancer cells (Additional file 1: Figure S1A). LC3 expression positively showed significant correlations with ALDH1, CD44, and CD133 expression (*R* = 0.849 with *P* < 0.001, *R* = 0.309 with *P* < 0.01, and *R* = 0.289 with *P* < 0.01, respectively; Fig. 1b). To confirm the immunofluorescence results, we next compared LC3 expression between CSCs and bulk cells in human pancreatic cancer cell lines and primary tumor-derived cells. Pancreatic CSCs were enriched by sphere formation and were characterized by elevated expression of CSC markers and embryonic stem cell transcription factors (SOX2 and NANOG) that are involved in the regulation of CSC functions [27] (Fig. 1c), chemoresistance, and anti-apoptosis (Additional file 2: Figure S2A and S2B). Enriched CSCs harvested from spheres exhibited higher LC3-II expression levels than the bulk cells did (Fig. 1c). Transmission electron microscopy images also showed increased autophagosome-like double-membrane structures in PANC-1 CSCs as compared with the bulk cancer cells (Additional file 1: Figure S1B). These results demonstrate that autophagic activity is upregulated in pancreatic CSCs.

### High LC3/ALDH1 coexpression confers a poor prognosis in pancreatic cancer patients

With the purpose of understanding the prognostic association among autophagy, pancreatic CSCs, and patient survival, the expression levels of LC3 and ALDH1 were detected by immunofluorescence staining and the survival probability were analyzed using the Kaplan-Meier method. Neither LC3 expression nor ALDH1 expression showed a statistically significant correlation with patient outcomes (Additional file 3: Figure S3A and S3B). However, patients with high expression levels of LC3 and ALDH1 simultaneously in pancreatic tumor cells demonstrated worse overall survival (OS) and disease-free survival (DFS) (*P* = 0.046 and *P* = 0.008; Fig. 1d), suggesting that coexpression of LC3/ALDH1 is a more specific prognostic marker for pancreatic cancer than is expression of either gene alone. Patient characteristics in respect to LC3/ALDH1 coexpression are outlined in Table 1. High LC3/ALDH1 coexpression significantly correlated with survival and recurrent status (*P* = 0.001 and *P* = 0.000; Table 1). Multivariate analysis showed that high LC3/ALDH1 coexpression is an independent prognostic factor for OS and DFS (hazard ratio = 4.721 with *P* = 0.004 and hazard ratio = 6.597 with *P* = 0.000; Table 2).

**Table 1:** Correlation between LC3/ALDH1 coexpression and clinicopathological profiles

Characteristic	Low LC3/ALDH1	High LC3/ALDH1	*P*
Age, median (range)	67 (38–85)	64 (37–85)	
Sex			
Male	37 (68.5)	23 (59.0)	0.342
Female	17 (31.5)	16 (41.0)	
Tumor location			
Head	28 (51.9)	29 (74.4)	0.177
Neck	5 (9.3)	2 (5.1)	
Body/tail	12 (22.2)	4 (10.3)	
Uncinate process	9 (16.7)	4 (10.3)	
Tumor size			
< 3 cm	30 (55.6)	22 (56.4)	0.935
≥ 3 cm	24 (44.4)	17 (43.6)	
Lymph nodes			
Negative	30 (55.6)	16 (41.0)	0.167
Positive	24 (44.4)	23 (59.0)	
Margin status			
R0	41 (75.9)	25 (64.1)	0.462
R1	11 (20.4)	12 (30.8)	
R2	2 (3.7)	2 (5.1)	
Tumor grade			
Poorly diff.	11 (20.4)	6 (15.4)	0.345
Moderate diff.	25 (46.3)	24 (61.5)	
Well diff.	18 (33.3)	9 (23.1)	
Stage			
I	10 (18.5)	1 (2.6)	0.097
II	40 (74.1)	36 (92.3)	
III	3 (5.6)	1 (2.6)	
IV	1 (1.9)	1 (2.6)	
CA19-9			
< 37U/ml	12 (22.2)	8 (20.5)	0.843
≥ 37U/ml	42 (77.8)	31 (79.5)	
Adjuvant therapy			
Yes	26 (48.1)	11 (28.2)	0.053
No	28 (51.9)	28 (71.8)	
Diabetes mellitus			
Yes	18 (33.3)	11 (28.2)	0.598
No	36 (66.7)	28 (71.8)	
Survival			
Yes	35 (64.8)	12 (30.8)	**0.001**
No	19 (35.2)	27 (69.2)	
Recurrence			
Yes	26 (48.1)	35 (89.7)	**0.000**
No	28 (51.9)	4 (10.3)	

The bold value indicates *P* < 0.05

**Table 2:** Outcomes of assessable patients with pancreatic cancer according to protein expression

Variable	Overall survival			Disease-free survival		
	HR	95 % CI	*P*	HR	95 % CI	*P*
LC3						
Low (*n* = 50)	1	0.087–0.589	0.200	1	0.251–1.387	0.226
High (*n* = 43)	0.227			0.590		
ALDH1						
Low (*n* = 51)	1	0.516–3.684	0.522	1	0.315–1.481	0.335
High (*n* = 42)	1.378			0.684		
LC3/ALDH1						
Low (*n* = 54)	1	1.622–13.739	**0.004**	1	2.535–17.170	**0.000**
High (*n* = 39)	4.721			6.597		
OPN						
Low (*n* = 58)	1	0.233–2.430	0.634	1	0.442–4.234	0.587
High (*n* = 35)	0.752			1.368		
OPN/LC3/ALDH1						
Low (*n* = 42)	1	Reference		1	Reference	
Moderate (*n* = 28)	1.770	0.846–3.704	0.130	1.351	0.773–2.362	0.291
High (*n* = 23)	2.385	1.129–5.038	**0.023**	2.245	1.183–4.259	**0.013**
OPN/CD44/CD133						
Low (*n* = 39)	1	Reference		1	Reference	
Moderate (*n* = 42)	1.929	0.897–4.146	0.093	1.639	0.889–3.021	0.113
High (*n* = 12)	4.527	1.403–14.604	**0.011**	4.140	1.672–10.254	**0.002**

The bold value indicates *P* < 0.05

### Autophagy blockade reduces pancreatic CSC activity

**Fig. 2:**
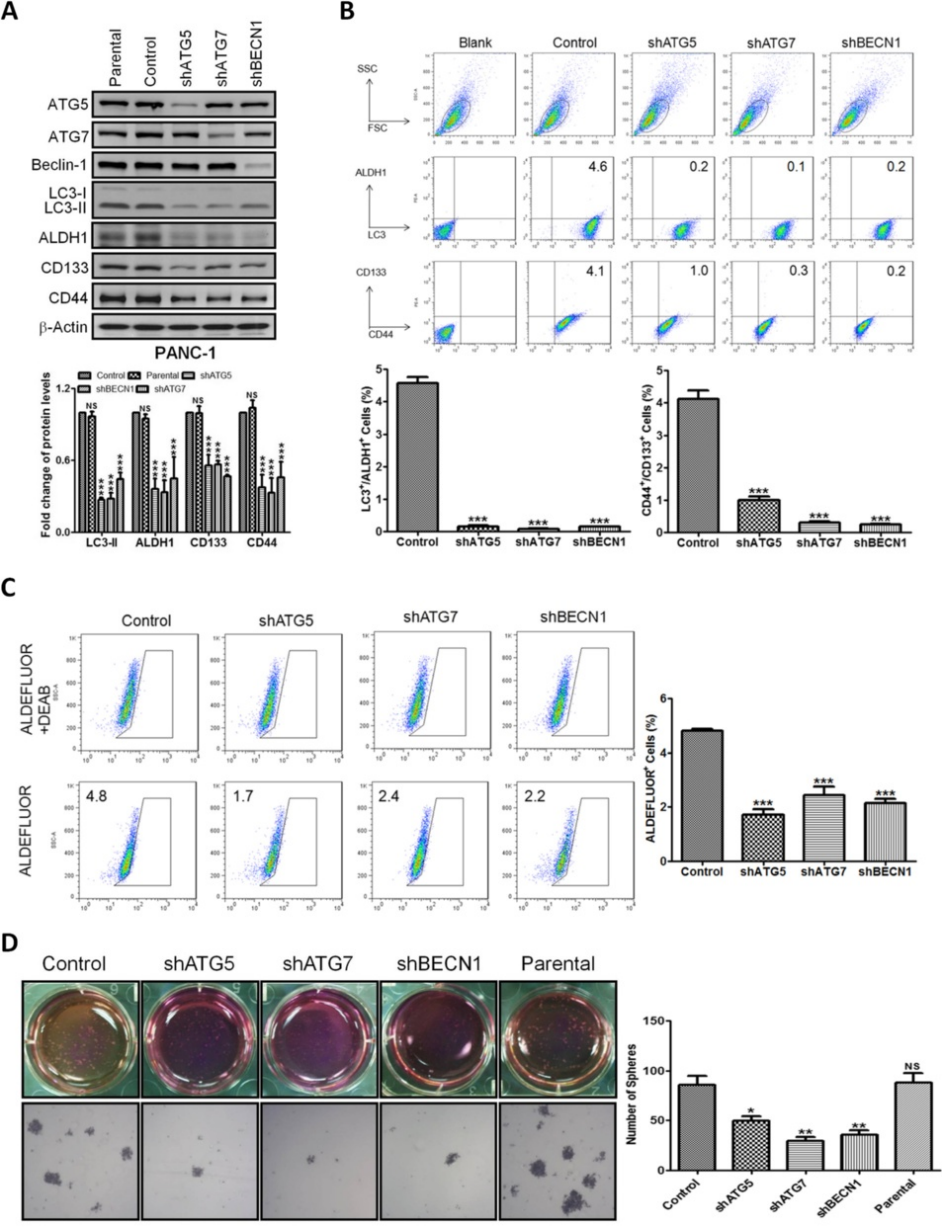
Blockade of autophagy by genetic knockdown reduces pancreatic CSC activity. **a** The expression of autophagy-related proteins and CSC markers in the PANC-1-derived cells permanently expressing shRNAs against *ATG5* (shATG5), *ATG7* (shATG7), and *BECN1* (shBECN1) was detected by Western blotting. The levels of LC3-II, ALDH1, CD133, and CD44, were normalized against β-Actin and presented as means ± SE of three separate experiments. NS, not significant; ***, *P* < 0.001, *vs.* control cells. **b** The expression of LC3, ALDH1, CD44, and CD133 in the control, shATG5, shATG7, and shBECN1 cells was analyzed by flow cytometry. The bar graphs indicate the percentage ± SE of LC3^+^/ALDH1^+^ cells (left) and CD44^+^/CD133^+^ cells (right). **c** The enzymatic activity of ALDH1 in the control, shATG5, shATG7, and shBECN1 cells was examined by ALDEFLUOR assay. The bar graph indicates the percentage ± SE of ALDEFLUOR^+^ cells in each cell line. **d** The bright field micrographs showed sphere formation of the control, shATG5, shATG7, shBECN1 cells cultured in ultra-low attachment plates for 14 days. The number of spheres was calculated and presented as means ± SE. NS, not significant; *, *P* < 0.05; **, *P* < 0.01; ***, *P* < 0.001 compared to controls

To determine the role of autophagy in pancreatic cancer stemness, we established lentivirus-mediated stable *ATG5*, *ATG7*, and *BECN1* knockdown cell lines shATG5, shATG7, and shBECN1 derived from PANC-1 cells and analyzed their CSC activity. Knockdown efficiencies were determined by Western blotting. The protein expression of CD44, CD133, and ALDH1 was found to be significantly decreased in shATG5, shATG7, and shBECN1 cells (Fig. 2a). Flow cytometry analysis showed that LC3^+^/ALDH1^+^ cells comprised 4.6 % of PANC-1 control cells; however, silencing *ATG5*, *ATG7*, and *BECN1* decreased the percentages of LC3^+^/ALDH1^+^ cells to 0.2, 0.1, and 0.2 %, respectively. Similarly, the percentages of CD44^+^/CD133^+^ cells were also reduced to 1.0 % in shATG5 cells, 0.3 % in shATG7 cells, and 0.2 % in shBECN1 cells when compared with 4.1 % in PANC-1 control cells (Fig. 2b). Because ALDH1 activity is a critical feature of CSCs, we performed the ALDEFLUOR assay to examine the effect of autophagy blockade on ALDH1 activity. In comparison with 4.8 % in PANC-1 control cells, the percentages of cells possessing ALDH1 activity was reduced to 1.7 % in shATG5 cells, 2.4 % in shATG7 cells, and 2.2 % in shBECN1 cells (Fig. 2c). To further examine whether autophagy blockade affects the self-renewal capacity of pancreatic CSCs, we performed sphere-forming experiments. The results showed that the number of spheres was significantly lower in shATG5, shATG7, and shBECN1 cells than in the control cells (Fig. 2d).

**Fig. 3:**
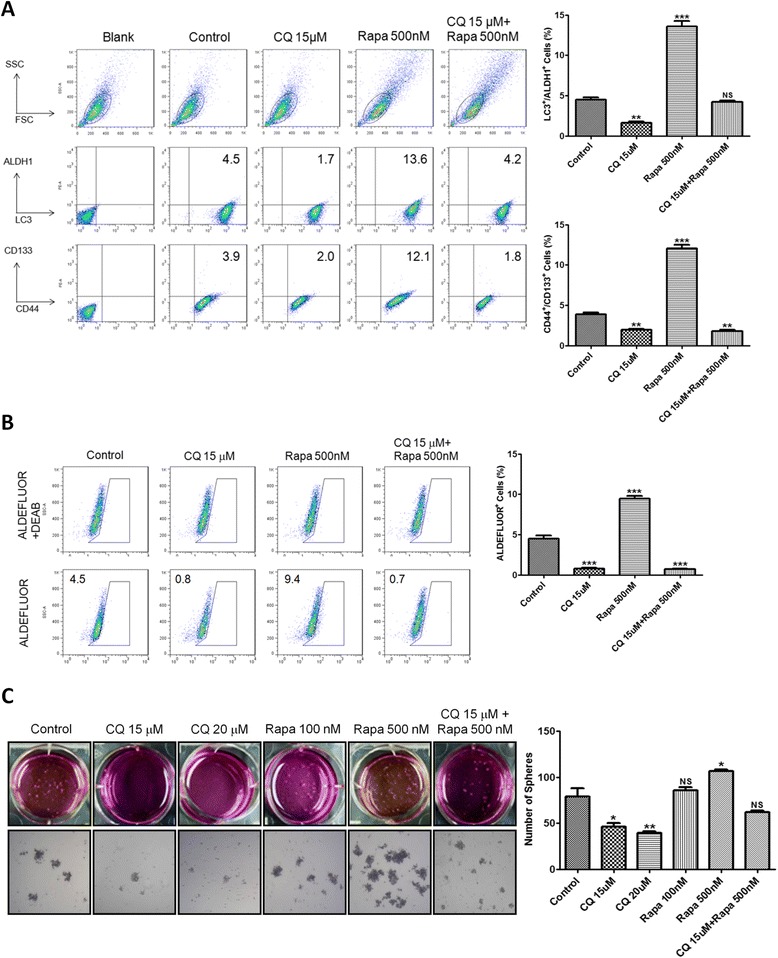
Pharmacological inhibition of autophagy reduces pancreatic CSC activity. **a** The expression of LC3, ALDH1, CD44, and CD133 in PANC-1 cells after 24 h of CQ, rapamycin (Rapa) and combination treatment was analyzed by flow cytometry. The graphs indicate the percentage ± SE of LC3^+^/ALDH1^+^ cells (upper) and CD44^+^/CD133^+^ cells (lower). **b** The enzymatic activity of ALDH1 in PANC-1 cells after 24 h of CQ, Rapa, and combination treatment was measured by ALDEFLUOR assay. The graph indicates the percentage ± SE of ALDEFLUOR^+^ cells in PANC-1 cells. **c** The bright field images reveal the sphere formation of PANC-1 cells after 14 days of culture in ultra-low attachment plates with CQ, Rapa, and combination treatment. The number of spheres was calculated and presented as mean ± SE. NS, not significant; *, *P* < 0.05; **, *P* < 0.01; ***, *P* < 0.001 compared to controls

To confirm the above-mentioned results, pharmacological manipulation of autophagy in pancreatic cancer cells was carried out. We tested whether interference with autophagy by autophagy inhibitor CQ and autophagy inducer rapamycin affects pancreatic CSC activity. Flow cytometry results showed that the percentage of LC3^+^/ALDH1^+^ cells was decreased from 4.5 % in untreated PANC-1 cells to 1.7 % by CQ treatment but was elevated to 13.6 % by rapamycin treatment. The percentage of CD44^+^/CD133^+^ cells was also decreased from 3.9 % in untreated PANC-1 cells to 2.0 % by CQ treatment but was increased to 12.1 % by rapamycin treatment (Fig. 3a). The results of ALDEFLUOR assay showed that CQ decreased the percentage of PANC-1 cells possessing ALDH1 activity in the corresponding control treatment group from 4.5 to 0.8 %, but rapamycin increased it to 9.4 % (Fig. 3b). Moreover, the number of PANC-1 spheres was decreased by CQ but was increased by rapamycin dose-dependently (Fig. 3c). Collectively, blockade of autophagy by both genetic and pharmacological inhibitors could markedly suppress pancreatic CSC activity, revealing the requirement of autophagy for CSC maintenance.

### Autophagy blockade prevents pancreatic cancer cell growth and tumor formation

**Fig. 4:**
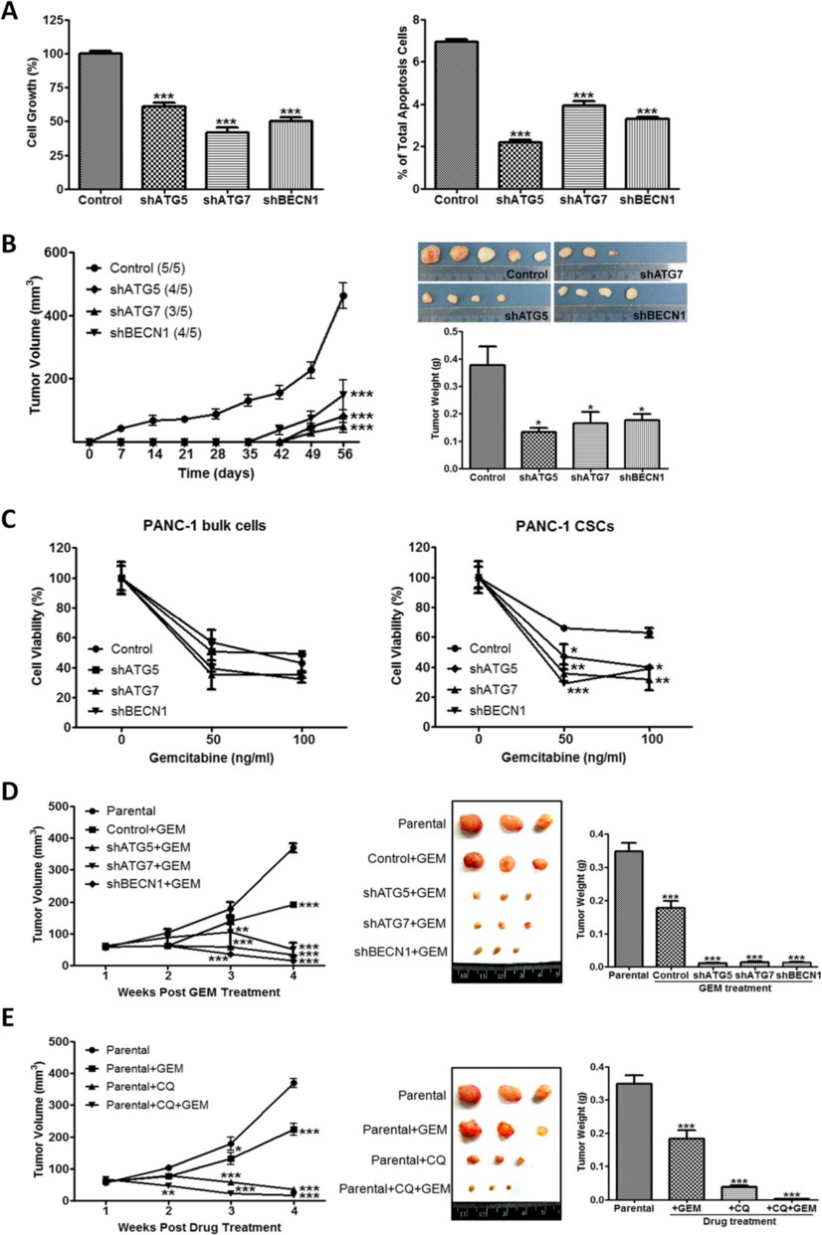
Blockade of autophagy by genetic knockdown suppresses tumor formation and sensitized pancreatic CSCs to gemcitabine. **a** The control, shATG5, shATG7 and shBECN1 cells were cultured for 48 h. Cell growth was analyzed by MTT assay (left panel). The percentages of apoptotic cells were determined by annexin V staining using flow cytometry (right panel). **b** The control, shATG5, shATG7, and shBECN1 cells were subcutaneously inoculated into the flanks of NOD/SCID mice. Each group contained 5 mice. The tumor volume was measured once weekly for 8 weeks, and the tumor weight was measured at the end of the experiment. **c** The bulk cells and the sphere-forming cells from the control, shATG5, shATG7 and shBECN1 cells were treated with gemcitabine for 48 h. The viability of the cells was determined by MTT assay. **d** The control, shATG5, shATG7, and shBECN1 cells were subcutaneously inoculated into the flanks of NOD/SCID mice. When the tumor volume reached 62.5 mm^3^, gemcitabine (GEM) was given intraperitoneally to mice at a dosage of 100 mg/kg once weekly for 4 weeks. **e** PANC-1 cells were subcutaneously inoculated into the flanks of NOD/SCID mice. When the tumor volume reached 62.5 mm^3^, mice were injected intraperitoneally with GEM (100 mg/kg), CQ (60 mg/kg), and their combination once weekly for 4 weeks. The tumor volume was measured every week after the first injection of each agent. The tumor weight in the lower panel was measured at the end of the experiment. Each group contained 3 mice. Values represent means ± SE. NS, not significant; *, *P* < 0.05; **, *P* < 0.01; ***, *P* < 0.001, significant differences between groups

To address the physiological significance of autophagy in the regulation of pancreatic CSC activity, we measured pancreatic cancer cell growth after autophagy was blocked. The MTT results showed that the cell growth of shATG5, shATG7, and shBECN1 cells was downregulated as compared with that of the control cells (Fig. 4a, left). We next determined whether blockade of autophagy leads to apoptosis and found that the number of apoptotic cells was not increased in shATG5, shATG7, and shBECN1 cells (Fig. 4a, right), suggesting that the decreased cell growth did not result from apoptosis. Because autophagy blockade decreased pancreatic cancer cell growth *in vitro*, we evaluated the effects of autophagy blockade on tumor progression *in vivo* using a NOD/SCID mouse xenograft model. Eight weeks after cell inoculation, xenografts from shATG5, shATG7, and shBECN1 cells had lower final tumor volume and tumor weight than did xenografts from the control cells (Fig. 4b), revealing a positive association between autophagic activity and pancreatic tumor growth rates.

### Autophagy blockade sensitizes pancreatic CSCs to gemcitabine

Since CSCs are believed to contribute to chemoresistance in cancer, we examined whether autophagy blockade affects gemcitabine resistance of pancreatic cancer cells. After treatment with gemcitabine, the cell survival rates of shATG5, shATG7, and shBECN1 cells were lower than that of the control cells though the results failed to show statistically significant difference between groups (Fig. 4c, left). We next isolated sphere-forming cells from the control, shATG5, shATG7, and shBECN1 cells to compare their resistance to gemcitabine. The cell viability of sphere-forming cells from shATG5, shATG7, and shBECN1 cells significantly decreased following gemcitabine treatment compared with that of the control cells (Fig. 4c, right). To confirm the *in vitro* results, pancreatic cancer cells were inoculated subcutaneously to NOD/SCID mice followed by the administration of gemcitabine when the tumor volume reached 62.5 mm^3^. Compared with a decline of 48 % in the tumor volume of mice inoculated with the control cells, gemcitabine could markedly reduce the tumor volumes by 91, 87, and 96 % in mice inoculated with shATG5, shATG7, and shBECN1 cells, respectively (Fig. 4d). Furthermore, a combined treatment of CQ and gemcitabine was more effective than was either agent alone in preventing pancreatic tumor formation (Fig. 4e). Therefore, autophagy blockade boosted the susceptibility of pancreatic CSCs to gemcitabine and thus enhanced the efficacy of gemcitabine against pancreatic cancer.

### OPN upregulates pancreatic CSC activity by activating autophagy

**Fig. 5:**
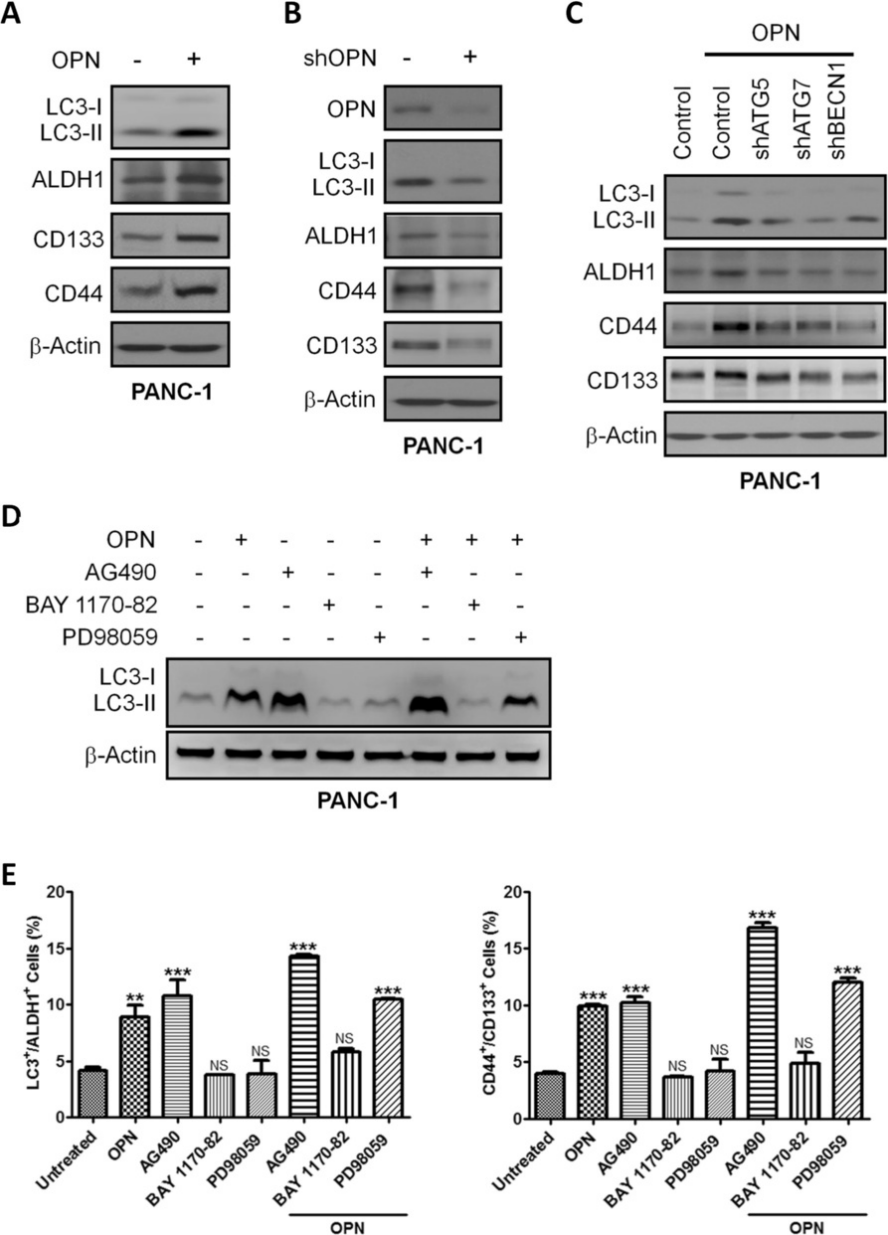
OPN upregulates pancreatic CSC activity by activating autophagy. **a** PANC-1 cells were treated with or without OPN (100 ng/mL) for 24 h. The lysates were collected for Western blotting using the indicated antibodies. **b** The lysates of PANC-1 cells with or without stable expression of *OPN*-specific shRNA were subjected to Western blotting using the indicated antibodies. **c** The control, shATG5, shATG7, shBECN1 cells were treated with OPN (100 ng/mL) for 24 h. The lysates were collected for Western blotting with the indicated antibodies. **d** and **e** PANC-1 cells were pretreated with the JAK2/STAT3 inhibitor AG490 (40 μM), the MEK/ERK inhibitor PD98059 (5 μM), and the NF-κB inhibitor BAY 1170–82 (5 μM) for 1 h followed by incubation with OPN (100 ng/mL) for 24 h. The lysates were subjected to Western blotting using LC3 antibodies. The number of LC3^+^/ALDH1^+^ cells and CD44^+^/CD133^+^ cells was measured by flow cytometry. Values represent means ± SE. NS, not significant; *, *P* < 0.05; **, *P* < 0.01; ***, *P* < 0.001 compared to controls

OPN has been reported to upregulate the sphere-forming capability of tumor cells [23, 24]. In addition, OPN can stimulate autophagy directly through the integrin/CD44 and p38 MAPK-mediated pathways in vascular smooth muscle cells [25]. Therefore, we hypothesized that OPN may upregulate CSC activity by activating autophagy. Confocal immunofluorescence results demonstrated that OPN treatment increased the number of ALDH1-expressing cells with upregulated LC3 puncta in PANC-1 cells, MIA PaCa-2 cells, and SP-1 primary pancreatic tumor cells. Blockade of autophagy by CQ led to LC3 puncta accumulation but prevented OPN-induced ALDH1 expression (Additional file 4: Figure S4A). Knockdown of *OPN* significantly suppressed cell growth (Additional file 3: Figure S3B), sphere formation (Additional file 4: Figure S4D), and tumor formation (Additional file 4: Figure S4E), but promoted apoptosis (Additional file 4: Figure S4C). The biochemical results showed that OPN induced LC3-II, ALDH1, CD44, and CD133 expression in PANC-1 cells (Fig. 5a), whereas knockdown of *OPN* decreased their expression (Fig. 5b). Blockade of autophagy by silencing *ATG5*, *ATG7*, and *BECN1* could hinder OPN-mediated upregulation of ALDH1, CD44, and CD133 (Fig. 5c). These findings demonstrated that OPN increased pancreatic CSC activity by activating autophagy. OPN exerts its biological functions by triggering multiple downstream signaling pathways, such as PI3K/AKT, NF-κB, MEK/ERK, STAT3, JNK, and p38 MAPK [21, 28, 29]. We therefore attempted to determine the major signaling pathway that mediates OPN-activated autophagy. In both PANC-1 and MIA PaCa-2 cells, the NF-κB, ERK, and STAT3 signaling pathways were activated by OPN, but AKT, JNK, and p38 MAPK were not (Additional file 5: Figure S5). To inhibit NF-κB, ERK, and STAT3 activation, the JAK2/STAT3 inhibitor AG490, the MEK/ERK inhibitor PD98059, and the NF-κB inhibitor BAY 1170–82 were used. Only BAY 1170–82 pretreatment could effectively block OPN-mediated increase in LC3-II expression (Fig. 5d) and the populations of LC3^+^/ALDH1^+^ cells and CD44^+^/CD133^+^ cells in PANC-1 cells (Fig. 5e), suggesting that OPN activated autophagy via NF-κB to increase pancreatic cancer stemness.

### Coexpression of OPN, LC3, and ALDH1 and coexpression of OPN, CD44, and CD133 portend poor patient outcomes

**Fig. 6:**
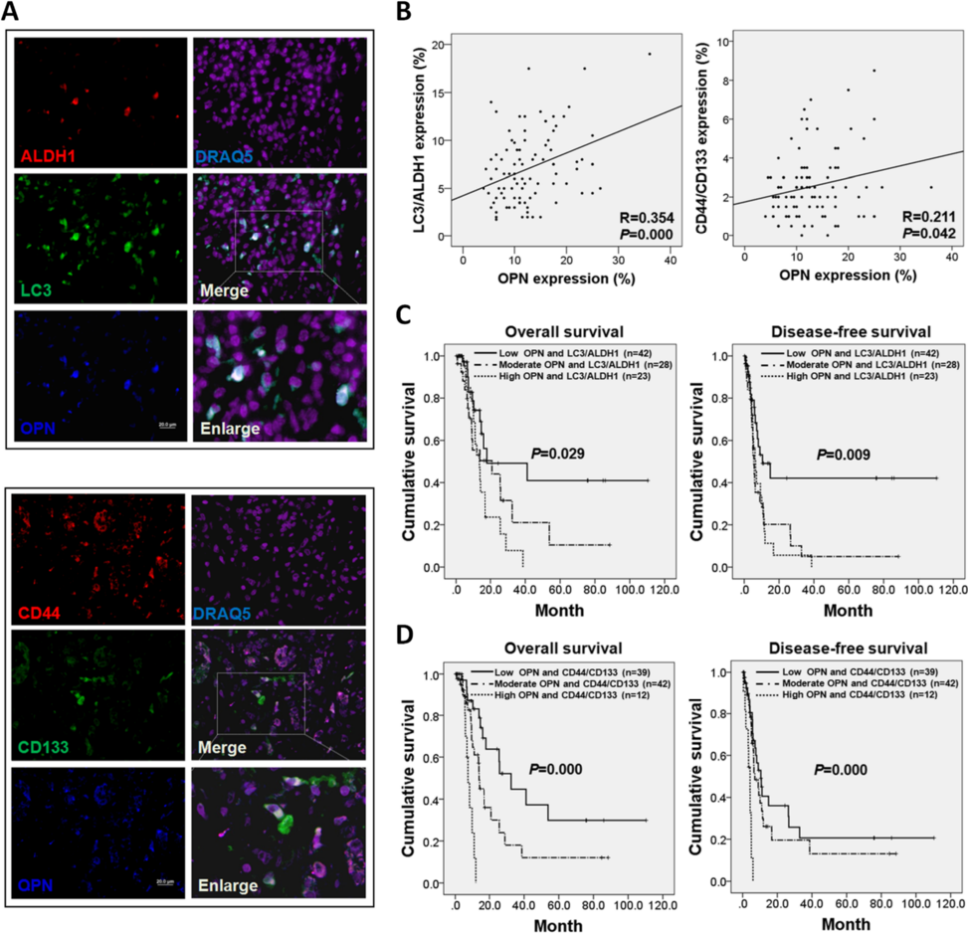
OPN/LC3/ALDH1 coexpression and OPN/CD44/CD133 coexpression are associated with poor patient survival. **a** Pancreatic cancer tissues were triplestained for OPN (blue), LC3 (red), and ALDH1 (green) (upper panel), and triplestained for OPN (blue), CD44 (red), and CD133 (green) (lower panel). The staining was visualized by confocal microscopy. Magnification: 400×, scale bar: 20 μm. **b** Immunofluorescence staining of OPN, LC3, ALDH1, CD44, and CD133 was performed in TMAs. OPN expression was positively correlated with LC3/ALDH1 coexpression (*R* = 0.354, *P* < 0.001) and CD44/CD133 coexpression (*R* = 0.211, *P* = 0.042). **c** Kaplan–Meier analysis showed that high levels of OPN/LC3/ALDH1 were associated with worse OS and DFS (*P* = 0.029 and *P* = 0.009). **d** Kaplan–Meier survival curves showed that high levels of OPN/CD44/CD133 were associated with worse OS and DFS (*P* < 0.001 and *P* < 0.001)

To assess the correlation of OPN with LC3/ALDH1 and CD44/CD133 coexpression in pancreatic tumor specimens, we performed triple immunofluorescence staining in TMAs. Confocal images revealed that OPN was highly expressed in both LC3/ALDH1-expressing cells and CD44/CD133-expressing cells (Fig. 6a, upper and lower, respectively). OPN expression showed a positive correlation with LC3/ALDH1 coexpression (*R* = 0.354 with *P* < 0.001) and CD44/CD133 coexpression (*R* = 0.211 with *P* < 0.05) (Fig. 6b). High levels of OPN/LC3/ALDH1 and OPN/CD44/CD133 were associated with poor OS (*P* = 0.029 and *P* = 0.000, respectively) and DFS (*P* = 0.009 and *P* = 0.000, respectively) (Fig. 6c, d). Multivariate analysis revealed that high OPN/LC3/ALDH1 coexpression and high OPN/CD44/CD133 coexpression were independently predictive for OS (hazard ratio 2.385 with *P* = 0.023 and hazard ratio 4.527 with *P* = 0.011, respectively) and DFS (hazard ratio 2.245 with *P* = 0.013 and hazard ratio 4.140 with *P* = 0.002, respectively) (Table 2).

## Discussion

CSCs are believed to drive the neoplastic progression, tumor recurrence, and metastasis. A comprehensive understanding of the biology of CSCs allows the development of effective therapies for pancreatic cancer. In this study, we have observed elevated levels of autophagy in pancreatic CSCs. Autophagy blockade profoundly reduced pancreatic CSC activity. OPN could induce autophagy by activating NF-κB and thus upregulate pancreatic CSC activity.

Recently, altered autophagy have been linked to CSCs. Dormant breast cancer stem-like cells induced by farnesyl transferase inhibitors expressed high levels of autophagy markers, such as ATG5, ATG7, and ATG12. Blockade of autophagy by 3-methyladenine decreased the proportion of stem-like cells [30]. The autophagic flux was upregulated in the sphere-forming breast cancer cells expressing ALDH1 compared with the bulk population. Knockdown of either *BECN1* or *ATG7* dramatically suppressed the sphere formation of breast CSCs [31]. In pancreatic cancer, cells with stem-like properties displayed stronger autophagic activity than did those with fewer stem cell markers. The enhanced autophagy enabled CSCs to survive under hypoxic stress [32]. Consistent with these reports, we observed that pancreatic CSCs exhibited elevated autophagy in both clinical specimens and cell lines. Blockade of autophagy by pharmacological or genetic inhibitors reduced CSC populations, sphere-forming ability, drug resistance, and tumor formation, revealing the requirement of autophagy for pancreatic CSC maintenance. Therefore, autophagy may act as a pro-survival regulator for pancreatic CSCs; however, the detailed mechanism is unclear. Similar to normal stem cells, CSCs have the ability to self-renew and differentiate through activation of several embryonic stem cell signaling pathways, including Wnt/β-catenin, Notch, and Hedgehog [33, 34]. In our study, when autophagy-related genes were knocked down in pancreatic cancer cells, β-catenin and Sonic Hedgehog expression was significantly decreased but Notch1 expression was not (Additional file 6: Figure S6), suggesting that autophagy may regulate pancreatic CSC activity by modulating Wnt/β-catenin and Hedgehog signaling.

Currently, CD44, CD133, and ALDH1 are the most commonly used markers in identification of pancreatic CSCs [35]. These markers, however, are not universal and perfect to identify CSCs because not all CSCs express the markers, and non-CSCs may also exhibit the markers. Therefore, the markers can be used for identification of CSC-rich subpopulations but not for unambiguous isolation of all of the CSCs. Likewise, autophagy may not be exclusively activated in CSCs but also in non-CSCs of pancreatic cancer. These may be the causes of the weak correlation between autophagy and single CSC marker expression (CD44 or CD133) in pancreatic tumor tissues. Since there is a partial overlap between CD44^+^/CD24^+^/ESA^+^ and CD133^+^ pancreatic cancer cells [6], it is conceivable that a combination of the markers may help to mark more pure CSCs than a single marker. Indeed, our group found that CD44^+^/CD133^+^ cells isolated from PANC-1 cells were capable of forming tumorspheres *in vitro*, exhibited tumor-initiating potentials *in vivo*, and profoundly responded to Wnt pathway activation or inhibition [36]. Accordingly, in our *in vitro* experiments, we used CD44 in combination with CD133 to identify a CSC population. We observed that the number of CD44^+^CD133^+^ cells was increased by the autophagy inducer rapamycin but was decreased by the autophagy inhibitor CQ or knockdown of autophagy-related genes, revealing the requirement of autophagy for the maintenance of CD44^+^CD133^+^ cells in pancreatic cancer. Unlike CD44 and CD133, ALDH1 showed a high correlation with LC3 expression in pancreatic tumors. This may be because ALDH1 expression and autophagy activation are regulated by some common signaling pathways. ALDH1 reportedly is a target gene of the NF-κB pathway [37–39]. Together with our observation that OPN triggered autophagy by activating NF-κB, the NF-κB pathway may act as an upstream positive regulator of both ALDH1 and autophagy in pancreatic cancer cells. However, further investigation is needed to substantiate our speculation.

The efficacy of current available therapeutic methods against pancreatic cancer remains limited. Gemcitabine is the standard chemotherapy used as first-line treatment for patients with advanced pancreatic cancer; however, the survival extension is only marginal [40, 41]. The high incidence of tumor relapse following treatment is believed to be caused by the presence of residual CSCs that are resistant to conventional therapies [42]. Impairment of CSC activity has been reported to reverse gemcitabine resistance [43, 44]. In this study, gemcitabine alone showed low efficacy in eliminating pancreatic CSCs and preventing xenograft tumor formation. However, blockade of autophagy by genetic knockdown or autophagy inhibitor CQ sensitized pancreatic CSCs to gemcitabine and thus almost eradicated xenograft tumors in mice. Our results are in good agreement with a previous study reporting that autophagy exerts a cytoprotective effect against chemotherapy drugs 5-FU and gemcitabine in pancreatic cancer cells, and inhibition of autophagy by CQ potentiated the growth-inhibitory effects of 5-FU and gemcitabine [18]. These findings highlight the exciting possibility that gemcitabine in combination with drugs that inhibit autophagy evokes a synergistic antitumor effect for pancreatic cancer treatment. Notably, a recent study demonstrated that CQ can counteract primary pancreatic CSC activity through inhibition of CXCR4 and Hedgehog signaling but independent of autophagy blockade, which is inconsistent with our result that, in primary pancreatic tumor SP-1 cells, CQ caused the accumulation of LC3 puncta, indicative of autophagy inhibition (Additional file 4: Figure S4A). CQ has also been reported to exert its actions not through inhibition of autophagy but through impairment of lysosomal functions [45]. Therefore, whether CQ-mediated inhibition of pancreatic CSC activity depends on its ability to block autophagy needs further confirmation.

OPN showed a positive association with coexpression of CD44/CD133 or LC3/ALDH1 in pancreatic tumors in this study; however the correlation was not strong. It is known that OPN exerts its biological functions through interaction with the integrin and CD44 families of cell surface receptors [20]. OPN-mediated functions may be determined by the different receptors. Therefore, analysis of OPN expression in combination with associated receptor expression in pancreatic cancer cells may be more appropriate to evaluate the correlation between OPN signaling and CSC activity than only OPN detection. Previously, OPN has been reported to stimulate autophagy in vascular smooth muscle cells [25]. We here found that OPN could also trigger autophagy via NF-κB in pancreatic cancer cells, and this finding is supported by several studies showing that NF-κB can act as an inducer of autophagy. For instance, autophagy was induced by NF-κB for cell survival during the heat shock response [46]. Upon hypoxia, ROS-activated NF-κB can trigger autophagy in breast cancer cells [47]. Intriguingly, autophagy seemed to be enhanced by the JAK2/STAT3 inhibitor AG490 in PANC-1 cells. Recently, STAT3 has been reported to suppress LC3 expression, thereby inhibiting autophagy and growth of pancreatic cancer cells [48]. Therefore, whether AG490 induces autophagy by inhibiting STAT3 activity or through its unknown off-target effects remained to be clarified.

## Conclusions

In this study, we provide evidence that OPN/NF-κB-mediated autophagy is required for pancreatic CSC maintenance, suggesting OPN/NF-κB/autophagy as an attractive target for eliminating pancreatic CSCs. Gemcitabine chemotherapy may offer greater therapeutic benefits to pancreatic cancer patients in combination with drugs against autophagy.

## Methods

### Cell lines and culture conditions

Human pancreatic cancer cell lines PANC-1, MIA PaCa-2, and AsPC-1 were obtained from American Type Culture Collection. All the cell lines and their derived cells were maintained in RPMI 1640 medium (Hyclone, SH30027.02) with 10 % fetal bovine serum (Invitrogen). Cells were incubated at 37 °C in a humidified atmosphere containing 5 % CO_2_.

### Isolation of primary tumor cells

Primary pancreatic tumor SP-1 cells were collected from the centrifugal sedimentation of ascites obtained from a 50-year old male patient. The histopathological examination of the patient’s tumor tissue revealed a well-differentiated ductal adenocarcinoma of the pancreas. Briefly, the bulk of ascites cells were seeded on culture flasks containing RPMI 1640 medium (Hyclone, SH30027.02) with 10 % fetal bovine serum (Invitrogen), 2 mM L-glutamine and 1 % penicillin/streptomycin (Caisson) at 37 °C in a humidified atmosphere with 5 % CO_2_. After two weeks, fibroblasts and stellate cells were removed by two rounds of serial enzymatic detachment with 0.05 % Trypsin/ EDTA (Life Technologies). The resulting population of cells was confirmed as cancer cells by tumor formation assay *in vivo*. For the experiments described here, SP-1 cells were used in passages 8 to 12. Immortalization of the isolated tumor cells was not necessary.

### Sphere formation and CSC harvest

Cells were seeded into 6-well ultra-low attachment plates (Corning, 3471) at a density of 4 × 10^4^ cells/well with serum-free medium containing 10 ng/mL basic FGF (PeproTech, 100-18B), 20 ng/mL EGF (PeproTech, AF-100-15), insulin-transferrin-sodium selenite media supplement (Sigma-Aldrich, 3116), and 0.4 % bovine serum albumin (Sigma-Aldrich, A7030). After 14 days of culture, the number of spheres was counted and the spheres were disaggregated into single cells with accutase (Invitrogen, A11105-01). The viable cells were counted by Eosin Y (Sigma-Aldrich, 230251) and cultured for further use.

### Measurement of cell viability

The growth or viability of cells was assessed with MTT reagent (Sigma-Aldrich, M2003). Cells were seeded into 96-well plates at a density of 4 × 10^3^/well. After treatments, MTT was added to each well (final concentration of 0.5 mg/mL) followed by incubation at 37 °C for 4 h. The supernatant was removed, and DMSO was added to dissolve the blue-purple crystals of formazan. The optical density of the samples was measured at a wavelength of 540 nm by spectrophotometer (Thermo Scientific, Multiskan EX).

### CSC identification and apoptosis detection

For CSC identification, 2 × 10^5^ cells were stained for ALDH1, CD44, and CD133 (antibodies are listed in Additional file 7: Table S1). After washing with PBS, cells were analyzed by flow cytometry (FACS Canto II).

For detection of apoptosis, specific binding of Annexin V to phosphatidylserine was performed by incubating the cells in binding buffer containing a saturating concentration of Annexin V-FITC (BD Biosciences, 556547) according to the manufacturer’s protocol. Following incubation, cells were pelleted and analyzed by flow cytometry.

### Tumor formation in NOD/SCID mice and chemoresistance test

1 × 10^6^ cells were subcutaneously inoculated into the right flank of 8-week old male NOD/SCID mice to form tumors. When the size of individual tumor reached 5 mm in diameter, animals were injected intraperitoneally with gemcitabine (100 mg/kg), CQ (60 mg/kg), and their combination once weekly for 4 weeks. Tumor volume was measured every week at the beginning of treatments using the formula: volume = *a*^2^ × *b*/2 where *a* is the major diameter and *b* is the minor diameter vertical to *a*. Animals were raised and cared for according to the guidelines set up by the National Science Council, ROC. The animal experiments were approved by the Institutional Animal Care and Use Committee.

### Lentiviral transduction and stable cell line generation

*OPN*, *ATG5*, *ATG7*, *BECN1*, and non-target short hairpin RNA (shRNA) vectors were purchased from the National RNAi Core Facility, Academia Sinica. Both plasmids pMD.2G and psPAX2 for lentiviral packaging were kind gifts from Dr. Mettling Clément (Institut de Génétique Humaine, CNRS UPR1142, Montpellier, France). The lentiviral particles were made by transfecting above-mentioned plasmids into 293 T cells and collected at 48 h post transfection. PANC-1 and MIAPaCa-2 cells were infected in the presence of 4 μg/mL polybrene (Sigma-Aldrich, AL-118). Puromycin (Sigma-Aldrich, P9620) was used for drug selection of infected cells to generate permanent cell lines.

### ALDEFLUOR assay

ALDH1 activity was detected by ALDEFLUOR assay kit (Stem Cell Technologies, 01700) according to manufacturer’s protocol. Briefly, cells were incubated in Aldefluor assay buffer containing ALDH1 substrate bodipy-aminoacetaldehyde (1 μmol/L per 1 × 10^6^ cells) at 37 °C for 50 min. As a negative control, a fraction of cells from each sample was incubated under identical condition in the presence of ALDH1 inhibitor diethylaminobenzaldehyde (15 μmol/L per 0.5 × 10^6^ cells). The cells were then subjected to FACS analysis.

### Cell lysis and Western blot analysis

The harvested cells were lysed in ice for 30 min with lysis buffer (Cell Signaling Technology, 9803). Lysates were cleared by centrifugation at 14,000 rpm for 10 min at 4 °C. Protein concentrations were measured by the Bradford assay (Bio-Rad Laboratories, 500–0006). For Western blot analysis, cell lysates were boiled for 5 min with sample buffer before being resolved in SDS–polyacrylamide gels. The proteins were transferred to PVDF membrane (Millipore, IPVH00010). The membrane was blocked with 5 % skim milk in TBST buffer (20 mM Tris-HCl, pH 7.4, 150 mM NaCl, 0.1 % Tween 20) for one hour and then stained with primary antibodies at 4 °C overnight followed by incubation with secondary antibodies (antibodies are listed in Additional file 7: Table S1). The binding of each antibody was detected using an enhanced chemi-luminescence kit (PerkinElmer, NEL105001EA). The signals were detected by X-ray films (Fuji, 47410 08399) or a UV transilluminator (UVP Ltd., BioSpectrum™ 500 Imaging System) and analyzed by the Gel-Pro Analyzer 4.0 software (Media Cybernetics).

### Immunofluorescence staining and analysis of clinical samples

A total of 93 pancreatic cancer patients who underwent resection in National Cheng Kung University Hospital (NCKUH) were included in this study that was approved by Institutional Review Board of NCKUH. Anonymous archived samples of human pancreatic cancer, including both normal and malignant tissues, were obtained from Human Biobank of NCKUH for TMA construction. Paraffin-embedded TMAs were cut into 5 μm-thick sections and stained with primary antibodies (listed in Additional file 7: Table S1) at 4 °C overnight followed by incubation with secondary antibodies (listed in Additional file 7: Table S1) at room temperature for 1 h. Cell nuclei were counterstained with DAPI (blue; Molecular Probes, D3571) or DRAG5 (purple; Abcam, ab108410). Fluorescence imaging was performed using a laser scanning confocal microscope (Olympus, Fluoview 1000), and the signals were quantified with Tissue-Quest software. The percentage of protein staining for each tumor specimen was classified into two staining grades according to the mean value of protein expression (the high grade represents ≥ mean; the low grade represents < mean). Tumor heterogeneity was evaluated using Pearson correlation coefficients (*r*) in two different cores from the same tumor blocks, as described previously [49].

### Statistics

Data are expressed as means ± SE from three independent experiments. Statistical analysis in this article was performed by one- or two-way ANOVA using Prism 5.0 software. The median survival was estimated using the Kaplan-Meier method. The association between studied variables was evaluated using Pearson’s correlation coefficient test in SPSS 17.0. Significance was set at *P* < 0.05.

Supplementary Materials and Methods are available as Additional file 8.

## Additional files

Additional file 1: Figure S1. Autophagy is activated in pancreatic CSCs. (A) Pancreatic tumor tissues were immunofluorescently stained for LC3 (green), LAMP1 (red), and SQSTM1/p62 (red). Images were taken at 800× magnification, and white scale bar indicates 20 μm. (B) Autophagosome-like double-membrane structures in the sphere-forming cells and the bulk cells from PANC-1 cells were visualized by transmission electron microscopy. Magnification: 10,000×, Scale bar: 2 μm. Images on the lower panel are high-magnification of the areas outlined by red squares. The bar graph indicates the means ± SE of the number of autophagosome-like double-membrane vesicles per cell counted on at least 20 cells.*, *P*<0.05, *vs.* bulk cells. (For detail, please see Additional file 8). (TIFF 3054 kb)

Additional file 2: Figure S2. Pancreatic CSCs enriched by sphere formation exhibit increased chemoresistance and anti-apoptotic activity. PANC-1, MIA PaCa-2, AsPC-1, and SP-1 cells were cultured in ultra-low attachment plates for 14 days to form spheres. The bulk cells and the sphere-forming cells were treated with gemcitabine for 48 h. (A) The viability of the cells was analyzed by MTT assay. (B) The percentages of apoptotic cells were determined by annexin V/PI staining. The Values represent means ± SE. *, *P*<0.05; **, *P*<0.01; ***, *P*<0.001, *vs.* bulk cells. (TIFF 1784 kb)

Additional file 3: Figure S3. Neither LC3 nor ALDH1 expression shows significant correlation with patient outcomes. (A) Kaplan–Meier analysis showed that LC3 expression was not associated with both OS and DFS of patients (*P* = 0.593 and *P* = 0.840). (B) Kaplan–Meier analysis showed that ALDH1 levels were not associated with both OS and DFS (*P* = 0.897 and *P* = 0.898). (TIFF 1956 kb)

Additional file 4: Figure S4. Knockdown of *OPN* inhibits CSC activity, cell growth, and tumor formation, but promotes apoptosis. (A) PANC-1, MIA PaCa-2, and SP-1 cells were treated with OPN (100 ng/mL), CQ (15 μM), or their combination for 24 h followed by being stained with antibodies against LC3 and ALDH1, and then were visualized by confocal microscope (original magnification: 200×, scale bar: 50 μm). The images on the lower are high-magnification of the areas outlined by white squares. Scale bar: 20 μm. (B) The non-silenced control cells and cells permanently expressing *OPN*-specific shRNA (shOPN cells) derived from PANC-1 and MIA PaCa-2 cells were cultured in ultra-low attachment plates for 14 days to form spheres. The number of spheres was calculated and presented as means ± SE. (C) The control and shOPN cells derived from PANC-1 and MIA PaCa-2 were cultured for 48 h. The growth of the cells was analyzed by MTT assay (left panel). The control and shOPN cells were grown for 48 h. The percentages of apoptotic cells were determined by annexin V/PI staining using flow cytometry (right panel). (D) The control and shOPN cells were subcutaneously inoculated into the flanks of NOD/SCID mice. Each group contains 5 mice. The tumor volume was measured once per week for 4 weeks, and the tumor weight was measured at the end of the experiment. All results represent mean ± SE. NS, not significant; *, *P*<0.05; **, *P*<0.01; ***, *P*<0.001 compared to control cells. (TIFF 3302 kb)

Additional file 5: Figure S5. OPN triggered STAT3, ERK, and NF-κB activation, but not AKT, JNK, and p38 MAPK in pancreatic cancer cells. (A) PANC-1 and MIA PaCa-2 cells were treated with OPN (100 ng/mL) for the indicated time points. The cell lysates were prepared and subjected to Western blotting using the indicated antibodies. (B) PANC-1 and MIA PaCa-2 cells were incubated with OPN (100 ng/mL) for the indicated time points. Cells were lysed and fractionated, and the levels of NF-κB p65 in both cytosolic and nuclear fractions were detected by Western blotting. β-Actin serves as a cytosolic marker, and LAMIN A/C serves as a nuclear marker. (For detail, please see Additional file 8). (TIFF 2417 kb)

Additional file 6: Figure S6. The β-Catenin and Sonic hedgehog signaling pathways are involved in regulation of pancreatic CSC activity by autophagy. The cell extracts were prepared from the control, shATG5, shATG7 and shBECN1 cells and subjected to Western blotting with the indicated antibodies. (TIFF 1146 kb)

Additional file 7: Table S1. List of antibodies used in this study. (DOCX 20 kb)

Additional file 8: **Supplementary Materials and Methods.**(DOCX 17 kb)

## Abbreviations

ALDH1: Aldehyde dehydrogenase 1
CSCs: Cancer stem cells
CQ: Chloroquine
DFS: Disease-free survival
OPN: Osteopontin
OS: Overall survival
shRNA: Short hairpin RNA
TMAs: Tissue microarrays
