# Supplementary figures and images for: Blockade of autophagy reduces pancreatic cancer stem cell activity and potentiates the tumoricidal effect of gemcitabine
Source: Mol Cancer. 2015 Oct 12;14:179. doi: 10.1186/s12943-015-0449-3 (PMC4603764; doi:10.1186/s12943-015-0449-3)

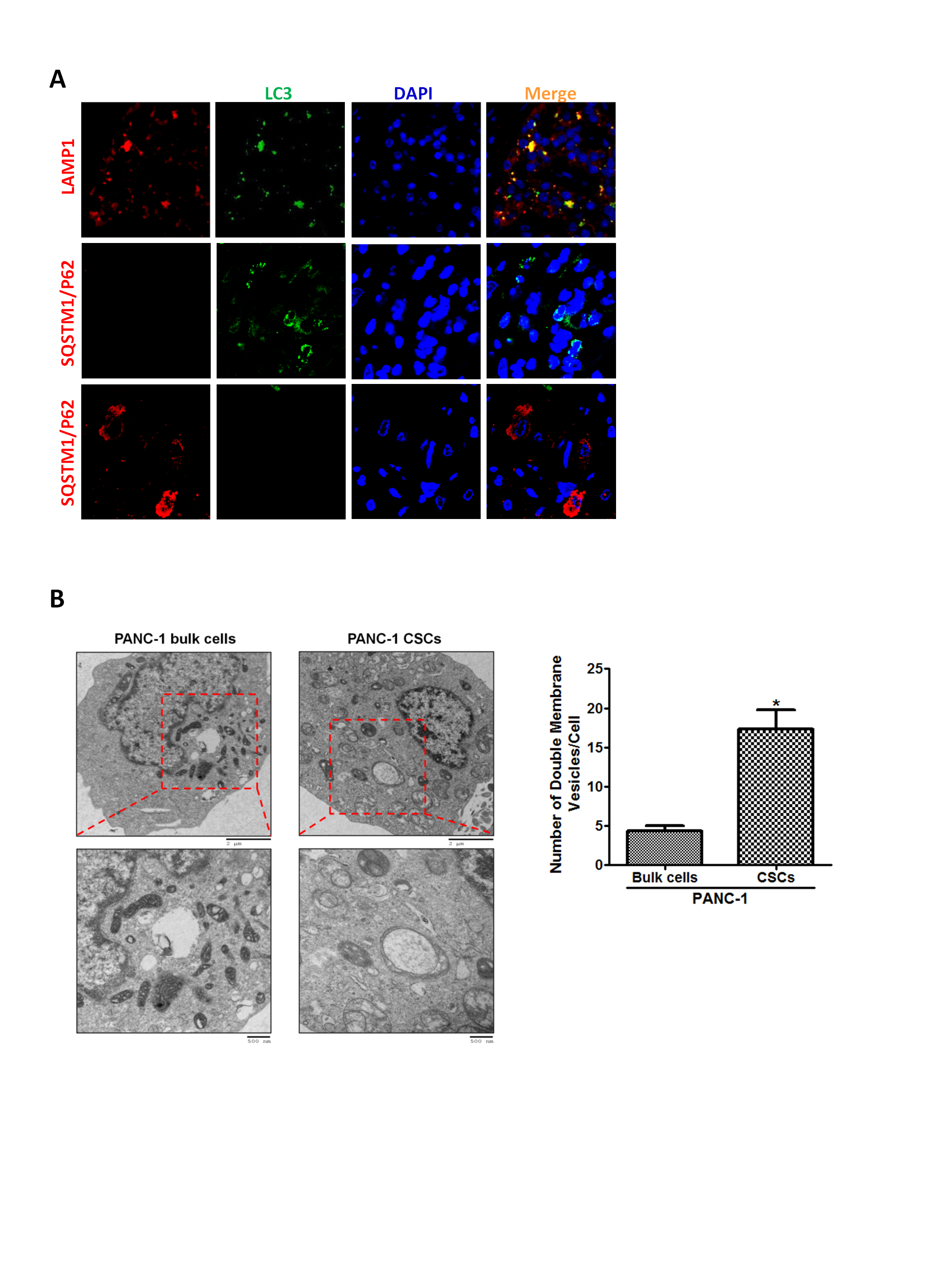

Supplement: Supplementary file 1 — Autophagy is activated in pancreatic CSCs. (A) Pancreatic tumor tissues were immunofluorescently stained for LC3 (green), LAMP1 (red), and SQSTM1/p62 (red). Images were taken at 800× magnification, and white scale bar indicates 20 μm. (B) Autophagosome-like double-membrane structures in the sphere-forming cells and the bulk cells from PANC-1 cells were visualized by transmission electron microscopy. Magnification: 10,000×, Scale bar: 2 μm. Images on the lower panel are high-magnification of the areas outlined by red squares. The bar graph indicates the means ± SE of the number of autophagosome-like double-membrane vesicles per cell counted on at least 20 cells.*, P<0.05, vs. bulk cells. (For detail, please see Additional file 8). (TIFF 3054 kb) [file 12943_2015_449_MOESM1_ESM.tif]

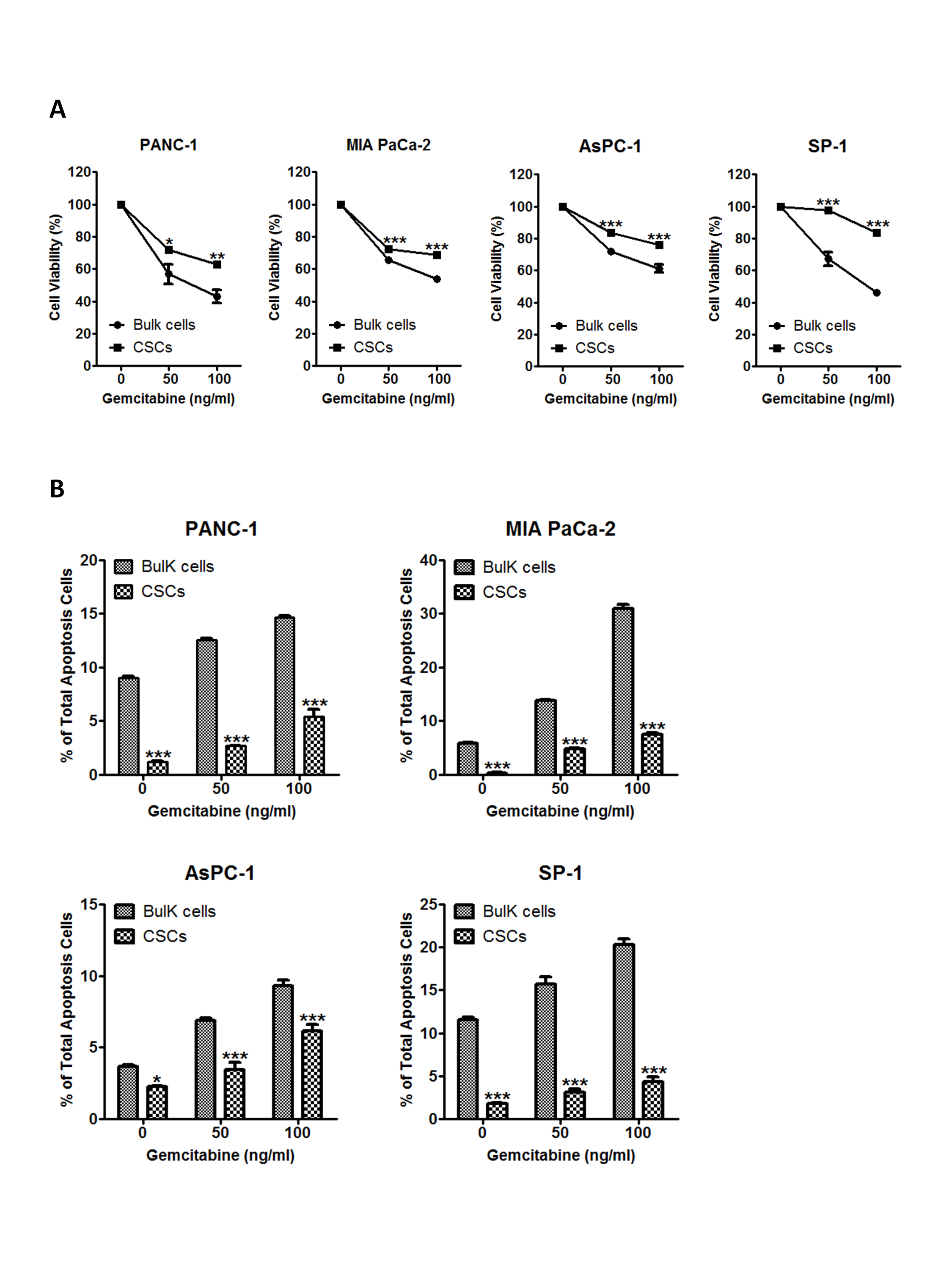

Supplement: Supplementary file 2 — Pancreatic CSCs enriched by sphere formation exhibit increased chemoresistance and anti-apoptotic activity. PANC-1, MIA PaCa-2, AsPC-1, and SP-1 cells were cultured in ultra-low attachment plates for 14 days to form spheres. The bulk cells and the sphere-forming cells were treated with gemcitabine for 48 h. (A) The viability of the cells was analyzed by MTT assay. (B) The percentages of apoptotic cells were determined by annexin V/PI staining. The Values represent means ± SE. *, P<0.05; **, P<0.01; ***, P<0.001, vs. bulk cells. (TIFF 1784 kb) [file 12943_2015_449_MOESM2_ESM.tif]

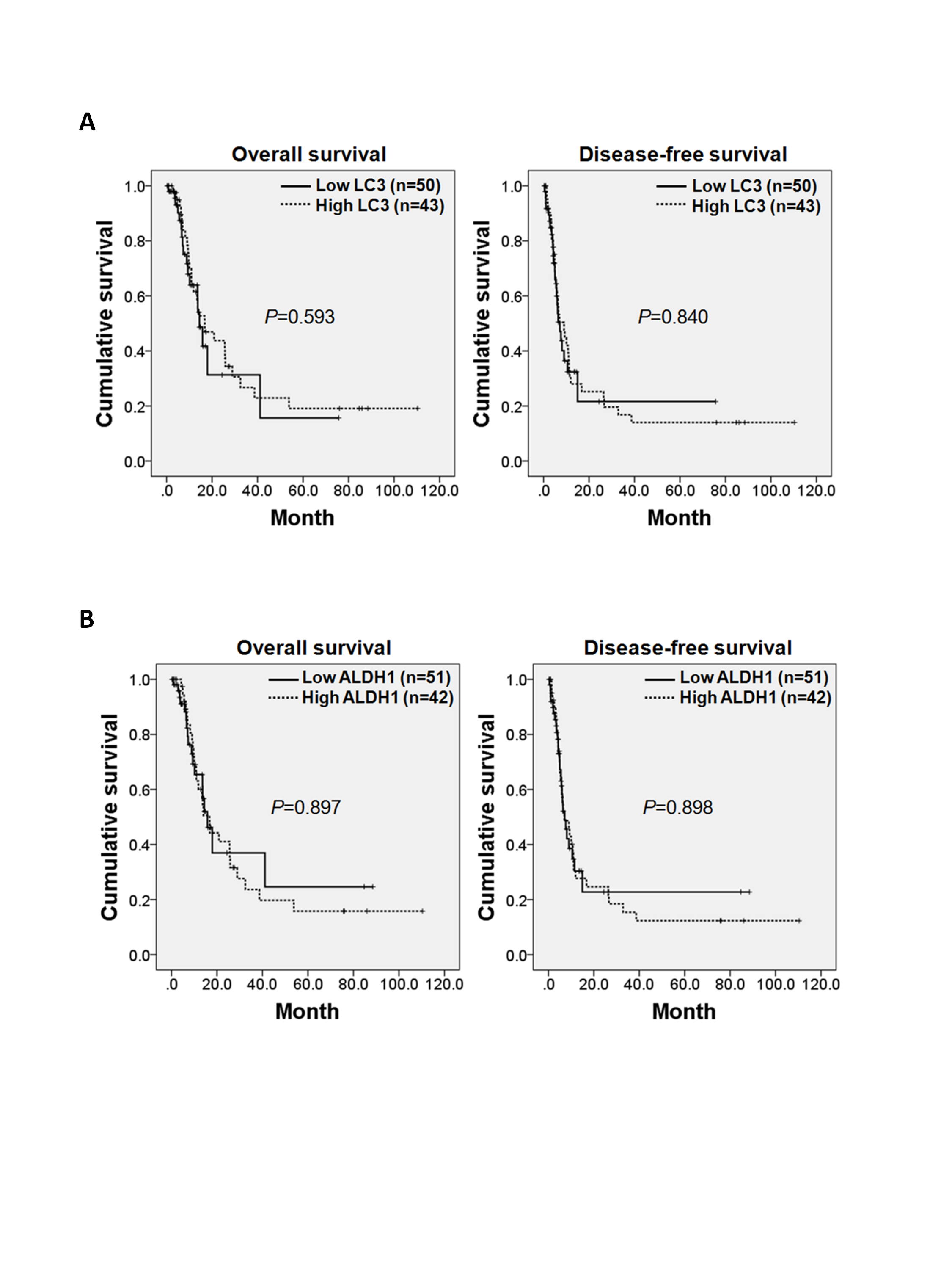

Supplement: Supplementary file 3 — Neither LC3 nor ALDH1 expression shows significant correlation with patient outcomes. (A) Kaplan–Meier analysis showed that LC3 expression was not associated with both OS and DFS of patients (P = 0.593 and P = 0.840). (B) Kaplan–Meier analysis showed that ALDH1 levels were not associated with both OS and DFS (P = 0.897 and P = 0.898). (TIFF 1956 kb) [file 12943_2015_449_MOESM3_ESM.tif]

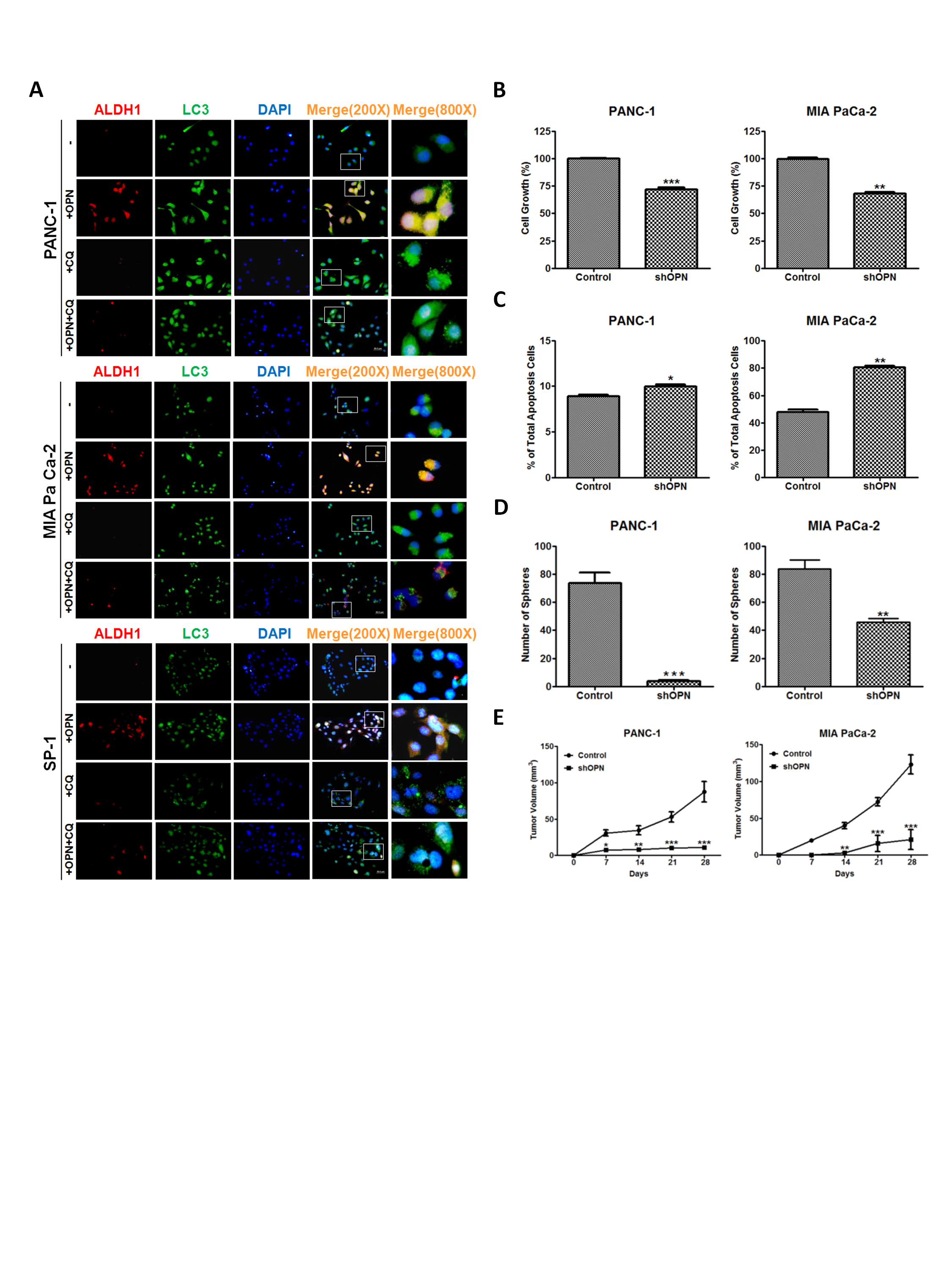

Supplement: Supplementary file 4 — Knockdown of OPN inhibits CSC activity, cell growth, and tumor formation, but promotes apoptosis. (A) PANC-1, MIA PaCa-2, and SP-1 cells were treated with OPN (100 ng/mL), CQ (15 μM), or their combination for 24 h followed by being stained with antibodies against LC3 and ALDH1, and then were visualized by confocal microscope (original magnification: 200×, scale bar: 50 μm). The images on the lower are high-magnification of the areas outlined by white squares. Scale bar: 20 μm. (B) The non-silenced control cells and cells permanently expressing OPN-specific shRNA (shOPN cells) derived from PANC-1 and MIA PaCa-2 cells were cultured in ultra-low attachment plates for 14 days to form spheres. The number of spheres was calculated and presented as means ± SE. (C) The control and shOPN cells derived from PANC-1 and MIA PaCa-2 were cultured for 48 h. The growth of the cells was analyzed by MTT assay (left panel). The control and shOPN cells were grown for 48 h. The percentages of apoptotic cells were determined by annexin V/PI staining using flow cytometry (right panel). (D) The control and shOPN cells were subcutaneously inoculated into the flanks of NOD/SCID mice. Each group contains 5 mice. The tumor volume was measured once per week for 4 weeks, and the tumor weight was measured at the end of the experiment. All results represent mean ± SE. NS, not significant; *, P<0.05; **, P<0.01; ***, P<0.001 compared to control cells. (TIFF 3302 kb) [file 12943_2015_449_MOESM4_ESM.tif]

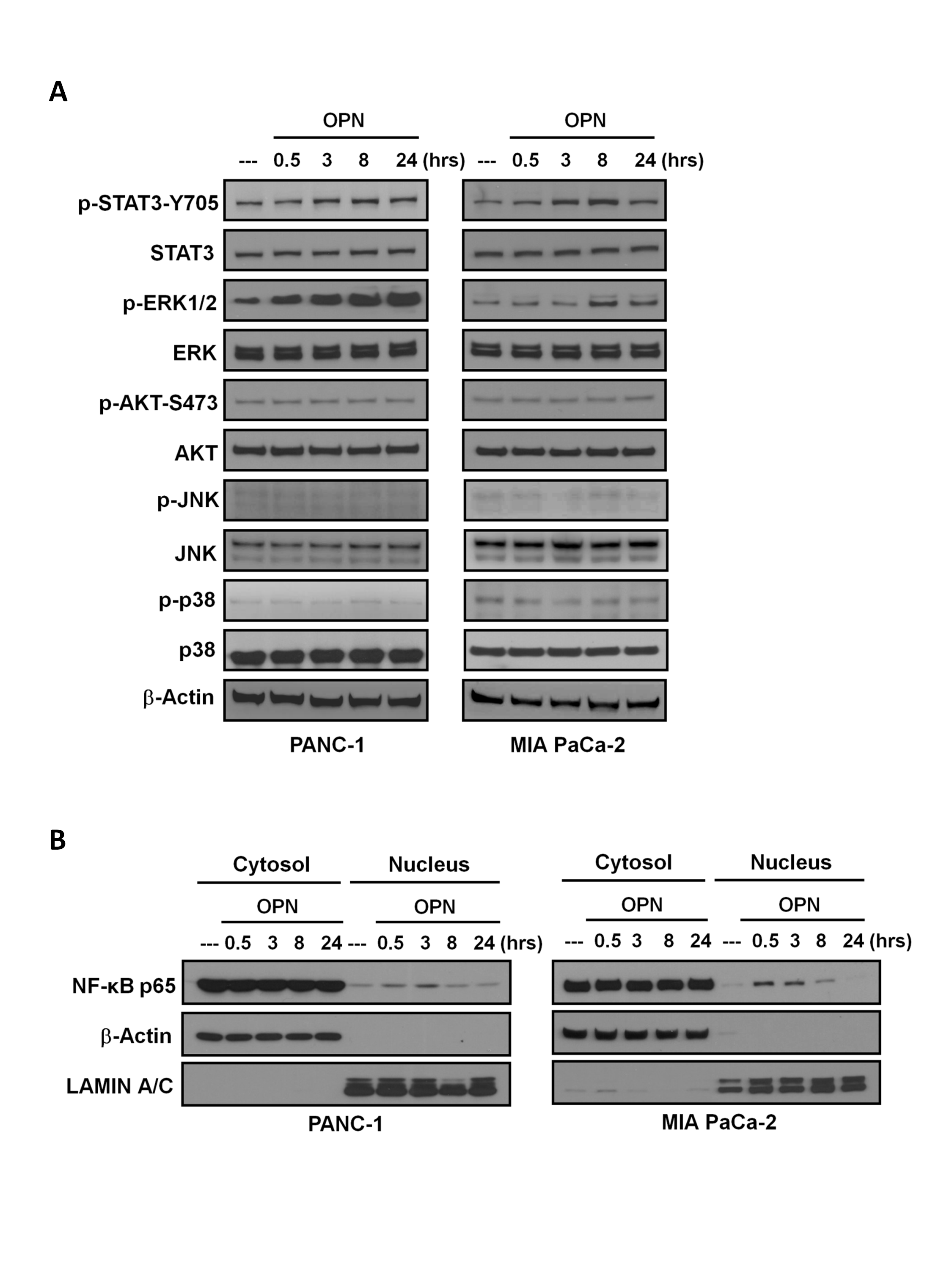

Supplement: Supplementary file 5 — OPN triggered STAT3, ERK, and NF-κB activation, but not AKT, JNK, and p38 MAPK in pancreatic cancer cells. (A) PANC-1 and MIA PaCa-2 cells were treated with OPN (100 ng/mL) for the indicated time points. The cell lysates were prepared and subjected to Western blotting using the indicated antibodies. (B) PANC-1 and MIA PaCa-2 cells were incubated with OPN (100 ng/mL) for the indicated time points. Cells were lysed and fractionated, and the levels of NF-κB p65 in both cytosolic and nuclear fractions were detected by Western blotting. β-Actin serves as a cytosolic marker, and LAMIN A/C serves as a nuclear marker. (For detail, please see Additional file 8). (TIFF 2417 kb) [file 12943_2015_449_MOESM5_ESM.tif]

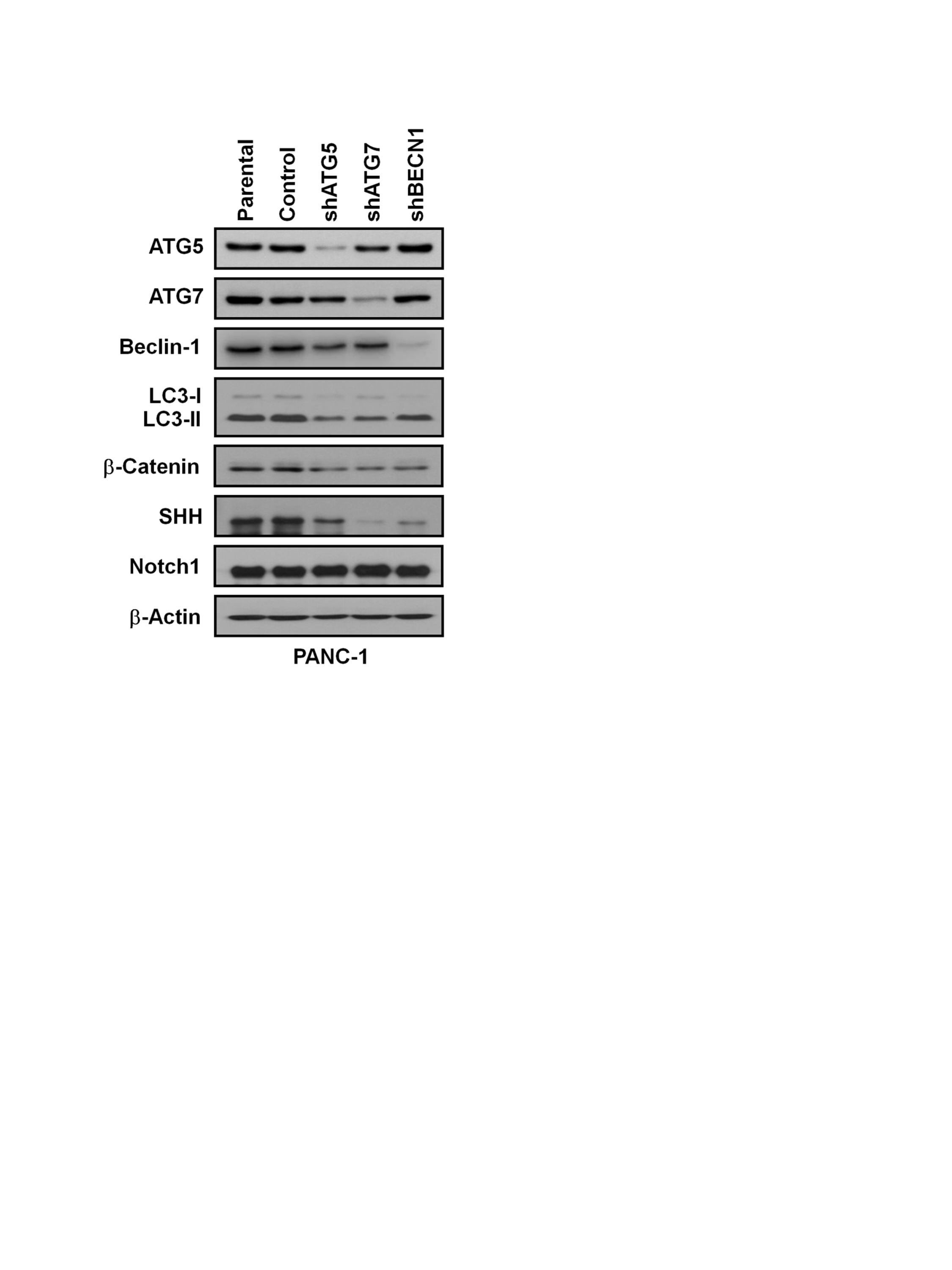

Supplement: Supplementary file 6 — The β-Catenin and Sonic hedgehog signaling pathways are involved in regulation of pancreatic CSC activity by autophagy. The cell extracts were prepared from the control, shATG5, shATG7 and shBECN1 cells and subjected to Western blotting with the indicated antibodies. (TIFF 1146 kb) [file 12943_2015_449_MOESM6_ESM.tif]
