# Supplementary material for: Blockade of autophagy reduces pancreatic cancer stem cell activity and potentiates the tumoricidal effect of gemcitabine
Source: Mol Cancer. 2015 Oct 12;14:179. doi: 10.1186/s12943-015-0449-3 (PMC4603764; doi:10.1186/s12943-015-0449-3)
Supplement: Supplementary file 7 — List of antibodies used in this study. (DOCX 20 kb) [file 12943_2015_449_MOESM7_ESM.docx]

**Supplementary Table S1. List of antibodies used in this study**

| **Primary antibodies** | | | | |
| --- | --- | --- | --- | --- |
| **Name** | **Catalog No.** | **Company** | **Application** | **Source** |
| Anti-β-Actin | MAB1501 | Millpore | WB (1:5000) | Mouse |
| Anti-ALDH1 | 611195 | BD Biosciences | FC (1:100)  IF (1:100)  WB (1:1000) | Mouse |
| Anti-AKT | #9272 | Cell Signaling Technology | WB (1:1000) | Rabbit |
| Anti-phospho-AKT (Ser473) | #9271 | Cell Signaling Technology | WB (1:1000) | Rabbit |
| Anti-ATG5 | #8540 | Cell Signaling Technology | WB (1:1000) | Rabbit |
| Anti-ATG7 | #8558 | Cell Signaling Technology | WB (1:1000) | Rabbit |
| Anti-Beclin-1 | sc-48381 | Santa Cruz | WB (1:1000) | Mouse |
| Anti-β-Catenin | #9562 | Cell Signaling Technology | WB (1:1000) | Rabbit |
| Anti-CD44 | 550392 | BD Biosciences | WB (1:2000) | Mouse |
| Anti-CD44 | M7082 | Dako | IF (1:500) | Mouse |
| Anti-CD44-FITC | 11-0441 | eBioscience | FC (1:10) | Rat |
| Anti-CD133 | 18470-1-AP | Proteintech | WB (1:1000) | Rabbit |
| Anti-CD133 | #3663 | Cell Signaling Technology | IF (1:500) | Rabbit |
| Anti-CD133-PE | 130-090-853 | Miltenyi Biotec | FC (1:10) | Mouse |
| Anti-ERK | #4695 | Cell Signaling Technology | WB (1:1000) | Rabbit |
| Anti-phospho-ERK (Thr 202/Tyr 204) | sc-136521 | Santa Cruz | WB (1:1000) | Mouse |
| Anti-LAMIN A/C | 612163 | BD Biosciences | WB (1:2000) | Mouse |
| Anti-LAMP1 | Ab25630 | Abcam | FC (1:200) | Mouse |
| Anti-LC3 | M152-3 | MBL | IF (1:100) | Mouse |
| Anti-LC3B | #2775 | Cell Signaling Technology | WB (1:1000) | Rabbit |
| Anti-NANOG | GTX100863 | GeneTex | WB (1:1000) | Rabbit |
| Anti-NF-κB p65 | 610869 | BD Biosciences | WB (1:1000) | Mouse |
| Anti-Notch1 | #3608 | Cell Signaling Technology | WB (1:1000) | Rabbit |
| Anti-OPN | Ab8448 | Abcam | WB (1:1000) | Rabbit |
| Anti-OPN | LS-B2411 | LifeSpan BioScience | IF (1:100) | Goat |
| Anti-SHH | #2207 | Cell Signaling Technology | WB (1:1000) | Rabbit |
| Anti-SOX2 | GTX101507 | GeneTex | WB (1:1000) | Rabbit |
| Anti-SQSTM1/p62 | GTX111393 | GeneTex | WB (1:2000)  IF (1:500) | Rabbit |
| Anti-STAT3 | 610190 | BD Biosciences | WB (1:2000) | Mouse |
| Anti-phospho-STAT3 (Tyr705) | #9131 | Cell Signaling Technology | WB (1:1000) | Rabbit |
| **Secondary antibodies** | | | | |
| **Name** | **Catalog No.** | **Company** | **Application** | **Source** |
| Anti-mouse IgG-HRP | 31430 | Thermo Scientific | WB (1:5000) | Goat |
| Anti-rabbit IgG-HRP | Ab6721 | Abcam | WB (1:5000) | Goat |
| Anti-mouse IgG-Alexa Fluor 488 | A11001 | Molecular Probes | IF (1:200) | Goat |
| Anti-mouse IgG-Alexa Fluor 568 | A11004 | Molecular Probes | FC (1:100)  IF (1:200) | Goat |
| Anti-rabbit IgG-Alexa Fluor 488 | A11008 | Molecular Probes | FC (1:100)  IF (1:200) | Goat |
| Anti-rabbit IgG-Alexa Fluor 568 | A11011 | Molecular Probes | IF (1:200) | Goat |
| Anti-goat IgG-Alexa Fluor 405 | Ab175664 | Abcam | IF (1:200) | Donkey |
