# Supplementary material for: Blockade of autophagy reduces pancreatic cancer stem cell activity and potentiates the tumoricidal effect of gemcitabine
Source: Mol Cancer. 2015 Oct 12;14:179. doi: 10.1186/s12943-015-0449-3 (PMC4603764; doi:10.1186/s12943-015-0449-3)
Supplement: Supplementary file 8 — Supplementary Materials and Methods.(DOCX 17 kb) [file 12943_2015_449_MOESM8_ESM.docx]

**Supplementary Data**

**Supplementary Materials and Methods**

**Preparation of nuclear and cytoplasmic extracts**

Cells were harvested by centrifugation at 1,500 rpm for 5 minutes, washed once with cold PBS, and resuspended in low salt permeabilization buffer (10 mM HEPES, pH 7.4, 10 mM KCl, 50 mg/ml digitonin) containing protease inhibitors (Roche Applied Sciences, 11873580001) for 30 minutes at 4°C followed by centrifugation at 3,000 rpm for 5 minutes. The supernatant was saved as cytosolic extract. The pallet was washed 3 times with low salt permeabilization buffer and then extracted with lysis buffer (Cell Signaling Technology, 9803) supplemented with protease inhibitors for 30 minutes at 4°C. After being clarified by centrifugation at 14,000 rpm for 10 minutes, the supernatant was saved as nuclear protein. The samples were analyzed by Western blotting.

**Transmission electron microscopy**

Cells were fixed with 2% paraformaldehyde and 2.5% glutaraldehyde for 30 minutes at room temperature followed by being post-fixed with 1% osmium tetroxide in 0.1 M sodium cacodylate buffer (pH 7.2) for one hour. The cells were then dehydrated through a graded series of increasing concentrations of ethanol (50%, 70%, and 100%) and propylene oxide. Next, the samples were embedded in epoxy resins (Epon, Fluka) and cut into ultrathin sections. The thin sections (80 nm) were collected on copper grids and stained with 5% uranyl acetate for 20 minutes and lead citrate for 10 minutes. The grids were analyzed by transmission electron microscopy (JEOL-1200, Tokyo, Japan) at an accelerating voltage of 80 kV.
